# Randomized Clinical Split-Mouth Study on Partial Ceramic Crowns Luted with a Self-adhesive Resin Cement with or without Selective Enamel Etching: Long-Term Results after 15 Years

**DOI:** 10.3290/j.jad.b4478817

**Published:** 2023-10-06

**Authors:** Julia L. Pfister, Marianne Federlin, Karl-Anton Hiller, Gottfried Schmalz, Wolfgang Buchalla, Fabian Cieplik, Konstantin J. Scholz

**Affiliations:** a Senior Dentist, Department of Conservative Dentistry and Periodontology, University Hospital Regensburg, Germany. Patient examination, analysis, wrote the manuscript.; b Associate Professor and Senior Dentist, Department of Conservative Dentistry and Periodontology, University Hospital Regensburg, Germany. Conceptualization, edited and proofread the manuscript.; c Mathematician and Statistician, Department of Conservative Dentistry and Periodontology, University Hospital Regensburg, Germany. Data analysis, edited and proofread the manuscript.; d Full Professor, Department of Conservative Dentistry and Periodontology, University Hospital Regensburg, Germany; Department of Periodontology, University of Bern, Bern, Switzerland. Conceptualization, edited and proofread the manuscript.; e Full Professor, Department of Conservative Dentistry and Periodontology, University Hospital Regensburg, Germany. Edited and proofread the manuscript.; f Associate Professor and Senior Dentist, Department of Conservative Dentistry and Periodontology, University Hospital Regensburg, Germany. Examined patients, wrote the manuscript. *These authors contributed equally and share senior authorship.

**Keywords:** partial ceramic crown, self-adhesive, enamel etching, long-term

## Abstract

**Purpose:**

This follow-up of a randomized clinical split-mouth study aimed to investigate the influence of selective enamel etching on the long-term clinical performance of partial ceramic crowns (PCCs) luted with a self-adhesive resin cement.

**Materials and Methods:**

43 patients received two PCCs (Vita Mark II; Cerec 3D) each for the restoration of extensive lesions with multiple cusp coverage, inserted with a self-adhesive resin cement (RelyX Unicem, RXU). Using a split-mouth design, one PCC received additional selective enamel etching (RXU+E) and one did not (RXU-E). Patients were clinically evaluated at baseline and after up to 15 years (median observation period 176 months) using modified USPHS and FDI criteria. The data were analyzed non-parametrically (chi-squared tests, α = 0.05). Clinical survival of all restorations after 15 years was evaluated using the Kaplan-Meier analysis.

**Results:**

After 15 years, 19 patients were available for clinical assessment (recall rate: 56%). Kaplan-Meier analysis showed a cumulative survival of 78.1% for RXU+E and of 42.9% for RXU-E, indicating a significantly higher survival rate for RXU+E (p = 0.004). Regarding the clinical performance of PCCs available for the 15-year evaluation, no statistically significant differences were found between RXU+E and RXU-E using modified USPHS and FDI criteria. Both groups revealed significant deterioration over time regarding surface luster, marginal adaptation, and marginal discoloration. RXU+E resulted in significantly inferior anatomic form over time and a significant improvement in post-operative hypersensitivity compared to baseline.

**Conclusion:**

For posterior PCCs, selective enamel etching can be recommended based on higher survival rates after 15 years. Clinically, deterioration due to aging is similar in both groups.

Tooth-colored partial-coverage restorations are widely used for the restoration of posterior teeth. They exhibit an estimated failure rate below 10% after 10 years and thus can be considered a clinical standard in contemporary dentistry.^28,47^ Partial ceramic crowns (PCCs) aim to combine three advantageous properties: functionality, esthetics, and reduced substance removal compared to full-coverage restorations for posterior teeth with extended tooth structure loss.^33^ The clinical success of a PCC is influenced by the condition of the remaining tooth structure and the quality of the ceramic restoration.^33^ The bond between restoration and prepared tooth structure as well as the preparation design may affect the overall survival of ceramic restorations and may eventually cause PCC failures in terms of fracture, debonding, and secondary carious lesions.^1,12^ Hence, a stable bond between the PCC and the dental hard tissues in addition to the resin-based composite (RBC) build-up restoration and the preparation design may be the most decisive factors for the long-term success of a PCC.^33^

An adequate bond can be achieved with various adhesives, which can often be time consuming and prone to error, due to rather high technique sensitivity in daily clinical practice.^6,29,32,33^ An adhesive that is easy to use and involves as few steps as possible would therefore be favorable. During the last two decades, several self-adhesive resin cements have been introduced on the market. Adhesion to enamel and dentin without or with smear layer may be achieved by chemical bonding between the resin cement and dental hard tissues, as known from glass-ionomer cements.^25^ Self-adhesion may additionally be achieved by micromechanical interactions, in particular with dentin.^38^ Previous in-vitro studies showed that the adhesion of self-adhesive resin cements to enamel was inferior compared to that of etch-and-rinse adhesives in combination with conventional resin cements.^22,24,46^ Therefore, additional selective enamel etching was proposed to create microretentions, enlarge the surface area, and increase the probability for micromorphological interactions with the enamel.^7,23,39^

Indirect ceramic CAD/CAM crowns, partial crowns, and inlays luted with three-step etch-and-rinse adhesives and conventional resin cements revealed high clinical survival rates of at least 82.4% over mid-term observation periods of 3 to 7 years.^15,19,40,41^ A systematic review and meta-analysis reported a survival rate of 91% for intracoronal glass and feldspathic ceramic restorations after 10 years.^28^ However, randomized clinical trials that investigate the clinical performance of PCCs luted with self-adhesive resin cements are rare. Clinical short- to medium-term data focussing on inlays over 2 and 4 years showed that the performance of self-adhesive resin cements was similar to that of conventional resin cements used in combination with adhesives,^31,43^ and additional selective enamel etching did not significantly improve the clinical performance of the restorations within a 4-year period.^31^ Previously, we reported the one-, two-, three-, and 6.5-year-results of a randomized clinical split-mouth study investigating the clinical performance and survival of PCCs luted with a self-adhesive resin cement (RelyX Unicem, 3M Oral Care, St Paul, MN, USA), with (RXU+E) or without (RXU-E) selective enamel etching.^3,13,36,37^ After 6.5 years of clinical service, we found a statistically significantly higher survival rate of PCCs inserted using the RXU+E protocol, but there were no differences between the two groups with respect to the clinical performance of the remaining restorations acceptable in terms of marginal adaptation or marginal staining, for instance.^3^ However, long-term data with observation periods >10 years are generally necessary for evaluating restorative procedures, since some complications, such as secondary caries lesions or restoration fractures, are more likely to appear at observation periods longer than five years.^11,18,30,45^

Therefore, the aim of the present follow-up investigation of a randomized clinical split-mouth study was to evaluate the clinical performance and the survival of PCCs luted with a self-adhesive resin cement with or without selective enamel etching after 15 years of clinical service. A second aim of this study was to compare modified USPHS and FDI criteria for evaluation of the clinical performance of PCCs after 15 years of clinical service. The null hypothesis tested was that selective enamel etching does not improve the long-term survival and clinical performance of PCCs luted with a self-adhesive resin cement.

## Materials and Methods

### Study Design

The present study is a 15-year follow-up examination of a prospective randomized controlled clinical split-mouth study, investigating the clinical performance and survival of PCCs luted with a self-adhesive resin cement (RelyX Unicem, 3M Oral Care; St Paul, MN, USA), with (RXU+E) or without (RXU-E) selective enamel etching.^36^ The one-, two-, three-, and 6.5-year results of this study have been published previously.^3,13,36,37^ The study design followed the requirements outlined in the CONSORT statement^27^ and the American Dental Association (ADA) Acceptance Program Guidelines for Tooth-Colored Restorative Materials for Posterior Teeth.^2^ The design of the original study and the present follow-up examination were approved by the Internal Review Board (IRB) of the University of Regensburg (references 06/092 and 11-65_1-101) in accordance with the 1964 Helsinki declaration and its later amendments or equivalent ethical standards. Written informed consent for this long-term investigation was obtained from all individual participants included in the study.

### Patient Recruitment

Forty-three (43) patients were been originally recruited from the patient pool of the Department of Conservative Dentistry and Periodontology at the University Hospital Regensburg. They had to present two defects (occlusal cavity width > 1/3 of the width in oro-vestibular direction) due to fractures, caries, or defective restorations in molars or premolars. Further criteria for inclusion are described in detail elsewhere.^3,13,36,37^

### Clinical Restorative Procedures

All clinical restorative procedures were performed by undergraduate students in the last year of their dental curriculum who were supervised by experienced dentists and trained with respect to preparation and fabrication of CAD/CAM PCCs. The procedures have been described in detail previously^3,13,36,37^ and thus are only reported briefly, as follows:

Existing restorations were removed and replaced by new resin-based composite build up-restorations (Clearfil New Bond & Clearfil Photo Core, Kuraray; Tokyo, Japan). When applicable, the pulpo-axial walls were lined with glass-ionomer cement (Ketac Bond, 3M Oral Care). Cavity preparations for PCCs were designed according to the size and extension of the defects to be restored following the current standards for preparation of PCCs published in the literature.^14,26^ Then, conventional dental impressions were taken using C-silicones (Silaplast & Silasoft, Detax; Ettlingen, Germany). Plaster cast models were prepared and subsequently scanned using the CEREC 3D system, version 3.0 (Dentsply Sirona; Bensheim, Germany), which was also employed for the design and manufacturing of the PCCs.

Temporary restorations (Luxatemp, DMG; Hamburg, Germany) were inserted using Temp-Bond NE (Kerr; Orange, CA, USA). In the meantime, PCCs were designed and milled using industrially prefabricated ceramic blocks (Vita Mark II, VITA Zahnfabrik; Bad Säckingen, Germany), which then were glazed. Temporary restorations were removed during the second appointment and PCCs were evaluated for adequate fit using a dental probe and silicon indicator paste (Fit Checker, GC; Tokyo, Japan). The PCCs were assigned to either the control group (RXU-E) or the test group (RXU+E) by coin toss. Following rubber-dam application, the two PCCs were inserted according to the respective luting procedure. The internal surfaces of the PCCs of both groups were etched with 5% hydrofluoric acid gel (Vita Ceramics Etch, VITA Zahnfabrik) for 60 s and silane (Monobond S, Ivoclar Vivadent; Schaan, Liechtenstein) was applied. The prepared teeth were cleaned with a slurry of pumice, rinsed with water and gently air dried.

In the RXU+E group, selective enamel etching was performed by applying 37% phosphoric acid gel (Total Etch, Ivoclar Vivadent) for 30 s followed by thorough rinsing with water for 30 s and gentle air drying. In the RXU-E group, no selective enamel etching was performed. For both groups, RXU was applied directly into the preparations and the PCCs were seated under constant digital pressure. Then, excess luting material was removed and the PCCs were light cured from the buccal, lingual (lower arch) or palatal (upper arch) and occlusal aspects for 40 s each with a halogen light curing unit (Elipar TriLight, 3M Oral Care) at an irradiance of 750 mW/cm^2^. The occlusion was adjusted and the PCCs were polished with Sof-Lex Contouring and Polishing Discs (3M Oral Care) and diamond polishing paste (Vita Karat; Vita Zahnfabrik).

### Clinical Examination

Clinical examination was performed by two blinded examiners out of a pool of trained and calibrated examiners (JLP, FC, KJS, and MF) who had not been involved in the respective treatments and were blinded regarding the luting procedure used for the respective PCC. The present study reports the results at baseline (BL) and 15 years, while previous studies have already reported the results up to 6.5 years.^3,13,36,37^ Whereas for these previous examinations, the modified USPHS criteria^34^ were employed, the present study used FDI criteria^21^ in addition to modified USPHS criteria.

All modified USPHS criteria (i.e., surface luster, color match, anatomic form, marginal adaptation, marginal discoloration, postoperative (hyper-)sensitivity, recurrent caries) were examined and scored as Alfa, Bravo, or Charlie. Alfa was defined as “success”, Bravo as “acceptable” and Charlie as “failure” for all USPHS criteria except “recurrent caries”. For “recurrent caries”, Alfa indicates clinical success and Bravo is defined as failure in terms of secondary caries. Likewise, all FDI criteria were recorded as follows:

- **Esthetic properties** Surface luster (A1)
  Surface staining (A2a)
  Marginal staining (A2b)
  Color match and translucency (A3; not examined at baseline investigation by USPHS criteria)
  Esthetic anatomical form (A4)
- **Functional properties**
  Fracture of material and retention (B5)
  Marginal adaptation (B6)
  Occlusal contour and wear (B7)
  Approximal anatomical form – contact point (B8a)
  Approximal anatomical form – contour (B8b)
- **Biological properties**
  Postoperative (hyper-)
  sensitivity and tooth vitality (C11)
  Recurrence of caries, erosion, abfraction (C12)
  Tooth integrity (enamel cracks, tooth fractures) (C13)
  Periodontal response (C14)

One of the following five scores was attributed to each criterion accordingly: clinically excellent/very good (1), clinically good (2), clinically adequate/satisfactory (3), clinically unsatisfactory (4), and clinically poor (5). Restorations rated with scores 1-3 were considered “clinically acceptable”, while restorations with scores 4 or 5 were considered “clinically unacceptable” and therefore regarded as a failure. Each restoration was examined independently by both examiners. In case of disagreement, a consensus between the investigators was reached by discussion while the patient was still present.

Postoperative hypersensitivity was determined by interviewing the patient and assessing tooth sensitivity using ice spray (Endo-Frost, Roeko, Coltène/Whaledent; Altstätten, Switzerland). Moreover, the papilla bleeding index (PBI^35^) was evaluated for assessing the patients’ oral hygiene level, and photographic documentation of each restoration was performed.

### Data Analysis

Data were analyzed using SPSS version 29 (IBM; Chicago, IL, USA) applying non-parametric statistical procedures. Kaplan-Meier survival rates were calculated based on the clinical failures that resulted in complete detachment or replacement of the restorations. For failure analysis, all those restorations were considered for which the file analysis showed when and why the PCCs had failed or whether the restorations were still in service. Five reasons for failure were distinguished: “debonding”, ”fracture”, “caries”, “endodontic treatment”, and “other reasons”. The log-rank test (Mantel-Cox) was used to test the equality of survival distributions for the different luting procedures (α = 0.05).

For clinical performance according to FDI or modified USPHS criteria, all examined patients with at least one restoration in service were included in the evaluation. Differences between luting procedures or over time (only available for modified USPHS criteria) were analyzed applying chi-squared tests (α = 0.05).

## Results

### Recall Rate

**Fig 1:**
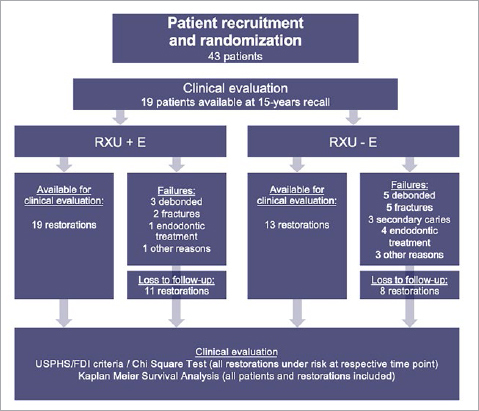
Flowchart showing the number of examined patients and restoration failures within groups at the respective time points.

Figure 1 shows the patient flow through the stages of this study. Out of initially 43 patients, 19 with 32 PCCs could be recruited for a further recall after a median (1st; 3rd quartile) observation period of 176 (175; 181) months or equivalent of 14.6 years. Eleven patients were male (57.9%) and 8 were female (42.1%). The median age of the available patients was 56 (range: 44 to 65) years at the present evaluation time point. In the 19 patients available for clinical evaluation, 19 PCCs inserted with RXU+E were still in service, while only 13 of 19 PCCs inserted with RXU-E were in service. Table 1 provides an overview of the distribution of restorations by localization.

**Table 1:** Distribution of the restorations regarding type of tooth and jaw

	RXU + E		RXU - E	
Tooth	4 premolars (21.1%)	15 molars (78.9%)	3 premolars (23.1%)	10 molars (76.9%)
Jaw	7 maxillary (36.8%)	12 mandibular (63.2%)	4 maxillary (30.8%)	9 mandibular (69.2%)

### Survival Analysis

Table 2 lists the reasons for failure that led to the replacement of the PCCs. The Kaplan-Meier survival analysis included 35 patients and 67 restorations (32 RXU+E, 35 RXU-E) for which it was evident from patients’ file analysis whether PCCs were still in service or when and why PCCs had failed. Three patients asked for study exclusion after recorded loss of the RXU-E restoration, so information on the respective RXU+E restoration performance and survival was not available at later recall appointments.

**Table 2:** Reasons for failure over time

Year of failure	Debonding		Fracture		Secondary caries		Endodontic treatment		Other reason	
	RXU+E	RXU-E	RXU+E	RXU-E	RXU+E	RXU-E	RXU+E	RXU-E	RXU+E	RXU-E
1	1	2	1	1	-	-	-	2	-	1
2	-	1	-	1	-	-	-	1	-	-
3	-	1	1	1	-	-	-	-	-	-
4	1	-	-	-	-	1	-	-	-	-
5	-	-	-	-	-	-	-	-	-	-
6	1	-	-	-	-	-	-	-	-	-
7	-	1	-	-	-	-	-	-	1	1
8	-	-	-	-	-	-	-	-	-	1
9	-	1	-	-	-	-	-	-	-	-
10	-	1	-	-	-	-	-	-	-	-
11	-	-	-	1	-	-	1	-	-	-
12	-	-	-	-	-	-	-	-	-	-
13	-	-	-	1	-	-	-	-	-	-
14	-	-	-	-	-	-	-	1	-	-
15	-	-	-	-	-	-	-	-	-	-
Total	3	7	2	5	-	1	1	4	1	3

**Fig 2:**
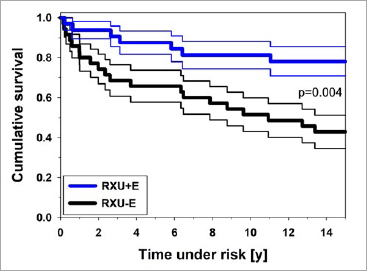
Cumulative survival of RXU+E und RXU-E after up to 15 years. The narrow lines indicate upper and lower confidence limits. RXU+E (blue): luting procedure with selective etching of enamel; RXU-E (black): luting procedure without selective etching of enamel.

The survival rates (95% confidence intervals) of the restorations at 15 years were 78.1% (± 7.3%) for RXU+E and 42.9% (± 8.4%) for RXU-E (Fig 2). The survival rates of RXU+E and RXU-E differed significantly (p = 0.004).

Within the first year after placement, debondings (n = 3), fractures (n = 2) and the need for endodontic treatment (n = 2) were reasons for failure. Within the first 5 years, the two main reasons for failure were debondings (n = 6) and fractures (n = 5). In contrast, reasons for failure such as caries lesions (n = 1), the need for endodontic treatment (n = 3) and “other reasons” were only found in a few isolated cases (n = 1).

### Clinical Performance

#### Clinical performance according to USPHS criteria

**Fig 3:**
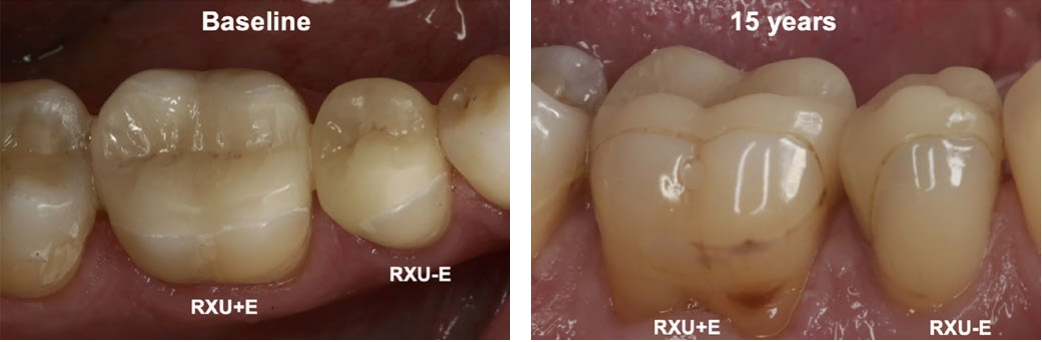
Clinical follow-up. Buccal view of teeth 45 (RXU-E) and 46 (RXU-E) at baseline and at the 15-year follow-up. Restoration 45 (RXU-E) reveals more distinct marginal discoloration involving a greater percentage of margin over time, but was given the same score as 46 using the FDI (score 3) and USPHS criteria (Bravo).

**Fig 4:**
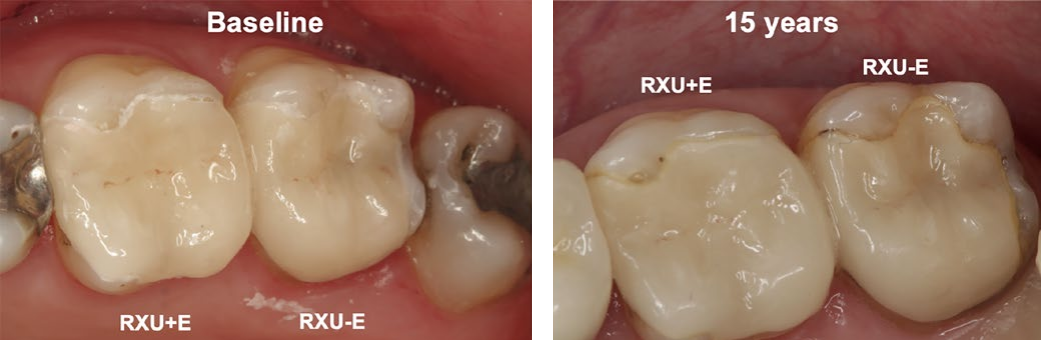
Clinical follow-up. Occlusal view of teeth 26 (RXU+E) and 27 (RXU-E) at baseline and 15 years. For both teeth, steps between the respective restoration and dental hard tissue slight marginal discolorations were visible at the 15-year follow-up. Restoration 27 (RXU-E) reveals more distinct marginal discoloration, rated with the same score as 26 using the FDI (score 3) and USPHS criteria (Bravo). Compared to the baseline examination, tooth 28 was extracted and tooth 25 received a new restoration.

Table 3 details the findings of the clinical evaluation according to modified USPHS criteria of all patients who could be recalled at 15 years and who presented with at least one restoration in service. Clinically, there were no significant differences between RXU+E and RXU-E at BL or after 15 years. However, there were significant differences over time between BL and 15-y within each group. Surface luster, marginal adaptation, and marginal discoloration showed a significant difference between BL and 15-y in both RXU+E and RXU-E (p ≤ 0.001), mostly due to an increase in Bravo ratings and decrease in Alfa ratings as a consequence of aging. For RXU+E, significant differences were also found for anatomic form (p = 0.034) and postoperative hypersensitivity (no hypersensitivity at 15 years, p = 0.001). Figures 3 and 4 show exemplary restorations for both groups at 15 years.

**Table 3:** Clinical performance according to USPHS criteria

USPHS criteria	Examination time point		USPHS score						Significant difference	
			RXU+E			RXU-E				
			Alfa	Bravo	Charlie	Alfa	Bravo	Charlie		
surface luster	BL	n	19	-	-	19	-	-	n.s.	
		%	100			100				
	15 y	n	4	15	-	6	7	-	n.s.	
		%	21.1	78.9		46.2	53.8		0.00	0.00
color match	BL	n	-	-	-	-	-	-	-	
		%								
	15 y	n	12	7	-	8	5	-	n.s.	
		%	63.2	36.8		61.5	38.5		-	-
anatomic form	BL	n	19	-	-	18	1	-	n.s.	
		%	100			94.7	5.3			
	15 y	n	15	4	-	11	2	-	n.s.	
		%	78.9	21.1		84.6	15.4		0.034	n.s.
marginal adaptation	BL	n	19	-	-	18	1	-	n.s.	
		%	100			94.7	5.3			
	15 y	n	1	18	-	2	11	-	n.s.	
		%	5.3	94.7		15.4	84.6		0.00	0.00
marginal discoloration	BL	n	19	-	-	19	-	-	n.s.	
		%	100			100				
	15 y	n	3	16	-	2	11	-	n.s.	
		%	15.8	84.2		15.4	84.6		0.00	0.00
postoperative hypersensitivity	BL	n	11	8	-	16	3	-	n.s.	
		%	57.9	42.1		84.2	15.8			
	15 y	n	19	-	-	13	-	-	n.s.	
		%	100			100			0.001	n.s.
recurrent caries	BL	n	19	-	-	19	-	-	n.s.	
		%	100			100				
	15 y	n	19	-	-	13	-	-	n.s.	
		%	100			100			n.s.	n.s.

Clinically acceptable scores = Alfa, Bravo. Non-acceptable scores = Charlie. p-values show significant differences between luting procedures at a respective examination time point or within one luting procedure over time. Dark grey: difference between RXU+E and RXU-E at the respective examination time points: light grey: difference between BL and 15 years, (left side: RXU+E, right side: RXU-E). n.s. = not statistically significant (p>0.05). – = no value.

#### Clinical performance according to FDI criteria

Table 4 shows the results of the clinical evaluation according to FDI criteria. There were no significant differences in any criterion between RXU+E and RXU-E, and all ratings for both RXU+E and RXU-E were within the clinically acceptable range.

**Table 4:** Clinical performance according to FDI criteria

FDI criteria	Examination time point		FDI score										Significant difference
			RXU+E					RXU-E					
			1	2	3	4	5	1	2	3	4	5	
A1 surface luster	15-y	n	4	13	2	-	-	5	8	-	-	-	n.s.
		%	21.1	68.4	10.5			38.5	61.5				
A2a surface staining	15-y	n	18	1	-	-	-	11	2	-	-	-	n.s.
		%	94.7	5.3				84.6	15.4				
A2b marginal staining	15-y	n	1	2	16	-	-	1	2	10	-	-	n.s.
		%	5.3	10.5	84.2			7.7	15.4	76.9			
A3 color match and translucency	15-y	n	10	9	-	-	-	4	9	-	-	-	n.s.
		%	52.6	47.4				30.8	69.2				
A4 esthetic anatomical form	15-y	n	16	2	1	-	-	11	2	-	-	-	n.s.
		%	84.2	10.5	5.3			84.6	15.4				
B5 fracture of material and retention	15-y	n	19	-	-	-	-	13	-	-	-	-	n.s.
		%	100					100					
B6 marginal adaptation	15-y	n	1	15	3	-	-	1	9	3	-	-	n.s.
		%	5.3	78.9	15.8			7.7	69.2	23.1			
B7 occlusal contour and wear	15-y	n	17	2	-	-	-	13	-	-	-	-	n.s.
		%	89.5	10.5				100					
B8a approx. anatomical form (contact point)	15-y	n	17	2	-	-	-	13	-	-	-	-	n.s.
		%	89.5	10.5				100					
B8b approx. anatomical form (contour)	15-y	n	18	1	-	-	-	13	-	-	-	-	n.s.
		%	94.7	5.3				100					
B10 patient’s view	15-y	n	19	-	-	-	-	13	-	-	-	-	n.s
		%	100					100					
C11 postop. (hyper-) sensitivity and tooth vitality	15-y	n	19	-	-	-	-	12	1	-	-	-	n.s.
		%	100					92.3	7.7				
C12 recurrence of caries, erosion, abfraction	15-y	n	19	-	-	-	-	12	1	-	-	-	n.s.
		%	100					92.3	7.7				
C13 tooth integrity (enamel cracks, tooth fractures)	15-y	n	2	16	1	-	-	5	7	1	-	-	n.s.
		%	10.5	84.2	5.3			38.5	53.8	7.7			
C14 periodontal response	15-y	n	2	12	5	-	-	1	8	4	-	-	n.s.
		%	10.5	63.2	26.3			7.7	61.5	30.8			
C15 adjacent mucosa	15-y	n	18	1	-	-	-	12	1	-	-	-	n.s.
		%	94.7	5.3				92.3	7.7				
C16 oral and general health	15-y	n	16	2	1	-	-	10	2	1	-	-	n.s.
		%	84.2	10.5	5.3			76.9	15.4	7.7			

Clinically acceptable scores (1–3) are highlighted in light blue, clinically non-acceptable scores (4–5) are highlighted in dark blue. There were no significant differences between luting procedures at the 15-y examination time point (dark grey fields); n.s. = not statistically significant (p>0.05). – = no value.

## Discussion

### Study Design

The aim of the present follow-up of a randomized split-mouth clinical trial was to evaluate the long-term clinical performance and survival of PCCs cemented with a self-adhesive resin cement with or without selective enamel etching after 15 years of clinical use. Additionally, evaluation criteria – modified USPHS and FDI criteria – for assessment of the clinical performance of PCCs after 15 years of clinical service were recorded.

In the present study, two PCCs per patient were investigated that had been randomly assigned to cementation with a self-adhesive resin cement applied either with or without selective enamel etching. The clinical study was designed as a prospective, randomized, controlled study which met the requirements of the CONSORT 2010 Statement^27,37^ and the requirements of the American Dental Association (ADA) Acceptance Program Guidelines for direct and indirect restorative materials (i.e., split-mouth design with at least 20 patients with two restorations each).^2^ The split-mouth design is particularly suitable for comparing two restorative procedures, e.g., the two luting procedures RXU+E and RXU-E, since factors such as oral hygiene and nutrition can be considered identical in the test and control restorations.^44^

Thanks to the inclusion of 43 patients at the beginning of the study, 19 patients could still be re-evaluated after 15 years. Because of the long observation period – which was not planned in the initial study proposal – some patients moved out of the region in the meantime and subsequently preferred to attend their local dentists. rather than accept long distances or additional travel costs to reach Regensburg, which may have led to an increased dropout rate over time, as discussed previously.^9^ In contrast to other studies, where all treatment steps were performed by one single, experienced dentist,^31,43^ in the present study, the restorations were performed by undergraduate dental students in their final semester under supervision of trained dentists. The heterogeneity of the practitioners as well as their practical experience can thus be factors influencing the clinical success of the PCCs, for example with regard to marginal adaptation or possibly anatomic form.^17^

### Kaplan-Meier Survival Rate

In the present study, PCCs cemented with RXU+E showed significantly higher survival rates due to lower failure rates in terms of debonding, fractures, and endodontic treatment. Over the 15-year period, a 78.1% survival rate was recorded for PCCs of group RXU+E and 42.9% for group RXU-E. The survival rates were significantly different (p = 0.004, Fig 2). Eleven PCCs inserted without selective enamel etching had failed during the first three years, in contrast to only 3 PCCs with selective enamel etching. The higher number of debonding failures in the RXU-E group within the first few years of clinical service may possibly be attributed to overdrying of the dentin during try-in and cementation under rubber-dam, which reduces the intrinsic wetness required for the cement reaction of the self-adhesive luting cement.^16^ Overdrying might be especially relevant with regard to the slower working pace of the dental undergraduate students compared to experienced practitioners. In contrast, rinsing off the acid in the selective etching procedure may involve the risk of accidentally etching the dentin, but also provides rewetting of the tooth structure prior to placement of the restorations.

Frankenberger et al^18^ reported two typical phase clusters of failure due to fractures in adhesively cemented all-ceramic inlay and onlay restorations: catastrophic failures during the first 3 to 4 years after insertion, which can be attributed to fatigue fractures caused by improper polishing of the ceramic, e.g. following occlusal adjustments, and fractures that occur after a prolonged period of over 10 years, mainly due to ceramic fractures at the restoration margins.

The fractures and debondings observed in the present study were mainly during the initial phase and thus caused by insufficient adhesion or deterioration of the bond.^13^ However, in contrast to the study by Frankenberger et al.,^18^ no second phase of failure could be detected yet in the present investigation. If restorations showed no failure during the first 5 years of clinical service, later failure occurred only occasionally (Table 2).

The null hypothesis – that there was no significant difference in survival of PCCs inserted with RXU+E and RXU-E – was rejected. After 15 years of clinical service, there was a significantly higher survival rate of the PCCs inserted with selective enamel etching, compared to the PCCs inserted without selective enamel etching.

### Clinical Performance Assessed by Modified USPHS Criteria

After 15 years in service, the results of the present clinical evaluation using modified USPHS criteria are still in line with the observations published for the clinical evaluation after 6.5 years.^3^

Concerning the criteria of surface luster, marginal adaptation, and marginal discoloration, there was significant deterioration in both groups over the 15-year period (in each case p < 0.001), represented by an increase in Bravo ratings as evaluated by USPHS criteria. In contrast, no significant decrease of surface luster scores had been noted after 6.5 years.^3^ Nevertheless, the decrease of surface luster scores is in line with the results of other studies,^31^ and can be attributed to occlusal contact wear and extrinsic mechanical wear.^5,31^

Marginal adaptation and marginal discoloration deteriorated significantly (p < 0.0001) over time for RXU+E and RXU-E. as indicated by USPHS criteria. The two criteria are linked.^10,18,31^ As self-adhesive resin cements showed more wear and filler debonding under load than did conventional resin cements in vitro, marginal discoloration due to the resulting formation of small gaps and surface inhomogeneities may be more likely to occur with self-adhesive resin cements.^4^ Also, because self-adhesive cement contains a certain amount of water, needed for creating an acidic pH at the moment of cementation in order to achieve adhesion, they are more prone to discoloration compared to resin-based materials. In the present study, those restorations that were still in service after 15 years of clinical service did not exhibit significantly better scores in marginal adaptation and discoloration when selective enamel etching was performed. However, this should not be overinterpreted, due to a preselection by a significantly higher failure rate of RXU-E restorations. The criterion “postoperative hypersensitivity” improved after 15 years compared to the baseline for both groups, and was statistically significant in group RXU+E (p = 0.001). This was already observed after 3 and 6.5 years,^3,13^ and confirmed the assumption that selective enamel etching could cause hypersensitivity, probably due to accidental etching of the dentin.^8,29,42,43^ However, the recorded hypersensitivity of the respective teeth decreased significantly within a short period of time. In general, it must be pointed out that significant clinical changes detected with either USPHS or FDI criteria were fully within the clinically acceptable range. After 15 years, no PCC available for evaluation had to be repaired or replaced due to insufficient clinical performance.

### Clinical Performance Assessed by FDI Criteria Compared to Modified USPHS criteria

Even without applying the sub-criteria, as was done in this study, the FDI criteria allow a more distinct subdivision into more scores, as reported in the literature.^26^ This is in line with a recently published revised version of the FDI criteria.^20^ Neverthless, neither with modified USPHS criteria nor with FDI criteria could statistically significant differences be detected between the two luting procedures RXU+E and RXU-E at 15 years.

When comparing USPHS and FDI scoring systems, in addition to improved discrimination within one criterion,^26^ the FDI criteria also provide a much more accurate description of the restorations being evaluated, as they include at most 17 criteria, whereas the USPHS criteria only include seven criteria. For example, the USPHS criterion “anatomic form” corresponds to three different FDI criteria, e.g., esthetic anatomical form, occlusal contour, and wear and approximal anatomical form. This enables the restoration to be described much more precisely in terms of its overall shape. In the case of clinical failure, the FDI criteria also provide a more accurate outcome, as the FDI criteria integrate an assessment of whether the restoration may be reparable or not. This was also implemented in the recently published revised FDI criteria, where specific FDI scores were linked to resultant treatment recommendations, such as reviewing and monitoring for scores 1-4, refurbishment or resealing for score 3, repair for score 4, and replacement for score 5.^20^

In summary, with both clinical evaluation methods, USPHS and FDI criteria, selective enamel etching had no significant effect on the clinical performance of PCCs luted with self-adhesive resin cements after 15 years of clinical service, although RXU+E restorations revealed a significantly higher overall survival rate compared to RXU-E.

## Conclusions

In spite of a low recall rate, PCCs luted with a self-adhesive resin cement can be recommended based on significantly higher survival rates after 15 years when additional enamel etching was performed compared to self-etching. At the 15-year follow-up, neither the modified USPHS criteria or the FDI criteria indicated significant differences regarding clinical performance between RXU+E and RXU-E restorations that were still in service. Both luting protocols showed a statistically significant decrease in surface luster and marginal adaptation over time as well as an increase in marginal discoloration.

## Clinical relevance:

Selective enamel etching prior to placement of ceramic partial crowns with self-adhesive luting cement resulted in higher survival after 15 years.
